# Supplementary material for: Modeling and application of production metering for electric pump wells without downhole pressure measurement devices
Source: PLoS One. 2025 Aug 20;20(8):e0330283. doi: 10.1371/journal.pone.0330283 (PMC12367175; doi:10.1371/journal.pone.0330283)
Supplement: S1. Table — Due to the fact that specific data involves oilfield commercial secrets and internal management information, this table only displays key parameters after desensitization processing, and is for reference only. [file pone.0330283.s001.docx]

S1 Table

| WELL_NAME | PROD_DATE | LIQ_PROD_DAILY | GAS_PROD_DAILY | DYNAMIC_LIQ_LEVEL | STATIC_LIQ_LEVEL | TUBING_PRES | FREQUENCE | I | V |
| --- | --- | --- | --- | --- | --- | --- | --- | --- | --- |
| well_1 | 2024/1/1 | 168 | 309 | 2213 | 1865 | 1.42 | 50 | 28 | 2064 |
| well_1 | 2024/1/2 | 162 | 309 | 2338 | 1886 | 1.43 | 50 | 28 | 2062 |
| well_1 | 2024/1/3 | 162 | 309 | 2332 | 1892 | 1.44 | 50 | 28 | 2072 |
| well_1 | 2024/1/4 | 164 | 309 | 2244 | 1804 | 1.44 | 50 | 28 | 2072 |
| well_1 | 2024/1/5 | 165 | 309 | 2232 | 1792 | 1.45 | 50 | 28 | 2072 |
| well_2 | 2024/1/1 | 123 | 71 | 1263 | 876 | 1.41 | 45 | 30 | 2528 |
| well_2 | 2024/1/2 | 123 | 83 | 1242 | 865 | 1.45 | 45 | 30 | 2528 |
| well_2 | 2024/1/3 | 123 | 83 | 1246 | 873 | 1.42 | 45 | 30 | 2516 |
| well_2 | 2024/1/4 | 125 | 82 | 1248 | 842 | 1.37 | 45 | 30 | 2521 |
| well_2 | 2024/1/5 | 125 | 83 | 1256 | 845 | 1.34 | 45 | 30 | 2521 |
| well_3 | 2024/1/1 | 42 | 0 | 1692 | 1107 | 1.36 | 50 | 32 | 1352 |
| well_3 | 2024/1/2 | 42 | 0 | 1685 | 1101 | 1.32 | 50 | 32 | 134 |
| well_3 | 2024/1/3 | 45 | 0 | 1686 | 1104 | 1.35 | 50 | 32 | 1351 |
| well_3 | 2024/1/4 | 44 | 0 | 1654 | 1083 | 1.33 | 50 | 32 | 1343 |
| well_3 | 2024/1/5 | 42 | 0 | 1672 | 1095 | 1.38 | 50 | 32 | 1350 |

S1 Table presents the parameters involved in the calculation process of the model in this article, which are extracted from the daily production data sheets of Tarim Oilfield. Due to the fact that specific data involves oilfield commercial secrets and internal management information, this table only displays key parameters after desensitization processing, and is for reference only.
