# Supplementary material for: Modeling and application of production metering for electric pump wells without downhole pressure measurement devices
Source: PLoS One. 2025 Aug 20;20(8):e0330283. doi: 10.1371/journal.pone.0330283 (PMC12367175; doi:10.1371/journal.pone.0330283)
Supplement: Attachment — Submitted filename: rebuttal letter.docx [file pone.0330283.s002.docx]

**Rebuttal Letter**

Manuscript Title: ***Modeling and application of production metering for electric pump wells without downhole pressure measurement devices***

Dear Editor and Reviewers,

We sincerely appreciate the time and effort you have dedicated to reviewing our manuscript. Your constructive comments have significantly improved the quality of our work. Below, we provide a point-by-point response to the reviewer’s suggestions, with corresponding revisions highlighted in the updated manuscript (v3-Revised Manuscript with Track Changes.docx).

**Response to Journal Requirements:**

**1.Formatting and File Naming Compliance**

We have strictly followed PLOS ONE's formatting templates and file naming requirements.

**2.Code Availability Statement**

This study does not involve any author-generated code, thus the code sharing policy is not applicable.

**3.Reference Verification**

We have thoroughly verified all references:

1. Ensured completeness (all citations match reference entries)
2. Confirmed no retracted papers were cited
3. Standardized formatting per PLOS ONE guidelines

**Response to Reviewer’s Comments**

**Comment 1**:*The description of the mathematical model could be slightly expanded to briefly outline key equations or assumptions.*

**Response**:

We have expanded the mathematical model description in **Section 1** to explicitly clarify the key assumptions and equations:

1. Added the assumption that "the ESP well production system operates under steady-state conditions, where the torque of the pump and the rotational speed of the motor remain constant", ensuring the validity of the energy conservation principle.
2. Highlighted the revised equation (Equation 1) incorporating the correction power term , which accounts for energy losses due to auxiliary components and aging.
3. Clarified the definition of the numerical constant 11.574 in Equation 2.
4. Added supplementary explanations for the motor output power calculations.

**Comment 2**:*It should be made clear how viscosity adjustment contributes to accuracy in the power calculation process.*

**Response**

In **Section 2.2**, the specific reasons for improving power calculation accuracy through viscosity correction are added, and a new reference is added to elaborate on the mechanism of viscosity influence on pump efficiency.

**Comment 3**:*The statement "calculated results align well with the metered results if periodically corrected" is vague. Consider specifying how frequently corrections are needed.*

**Response**:

We acknowledge that correction power variations may not follow linear time-dependent patterns. Therefore, instead of specifying fixed intervals, we recommend performing corrections as frequently as field conditions permit. Accordingly:

1. Removed the term "periodically" from the Abstract to avoid ambiguity.
2. Added clarification in **Section 4.1** (last paragraph).

**Comment 4**:*One crucial component of the model is how the pump performance curves are modified for viscous liquids. Give some thought to explaining the process by which these modifications were made and any restrictions on using them for certain fluid characteristics.*

**Response:**

We have made the following improvements:

1. Added a comprehensive viscosity correction flowchart (Figure 4) in **Section 2.2** to visualize the step-by-step procedure.
2. Regarding restrictions on fluid viscosity correction,we recognize this as a valuable suggestion for future research，but the ANSI standard does not explicitly specify limitations, so we do not provide any additional explanations in the original text.

**Conclusion**

We believe these revisions address all reviewer concerns and strengthen the manuscript’s technical rigor. Thank you again for your valuable feedback. Please let us know if further modifications are needed.

Sincerely,

[Sun Yizhen]
[Research Institute of Petroleum Exploration and Development]
[Phone: +86 13263398197]
[Email: 605182894@qq.com]
